# Preparation of Lambda-Cyhalothrin-Loaded Chitosan Nanoparticles and Their Bioactivity against *Drosophila suzukii*

**DOI:** 10.3390/nano12183110

**Published:** 2022-09-08

**Authors:** Rady Shawer, Eman S. El-Leithy, Rania S. Abdel-Rashid, Abdelazeem S. Eltaweil, Rowida S. Baeshen, Nicola Mori

**Affiliations:** 1 Department of Plant Protection, Faculty of Agriculture (Saba Basha), Alexandria University, Alexandria 21531, Egypt; 2 Department of Pharmaceutics and Industrial Pharmacy, Faculty of Pharmacy, October University for Modern Sciences and Arts (MSA), Cairo 12451, Egypt; 3 Department of Pharmaceutics and Industrial Pharmacy, Faculty of Pharmacy, Helwan University, Cairo 11795, Egypt; 4 Department of Chemistry, Faculty of Sciences, Alexandria University, Alexandria 21526, Egypt; 5 Department of Biology, Faculty of Science, Tabuk University, Tabuk 71421, Saudi Arabia; 6 Department of Biotechnology, Verona University, 37134 Verona, Italy

**Keywords:** ionotropic gelation, pyrethroids, tripolyphosphate, alginate, spotted wing drosophila

## Abstract

The encapsulation of pesticides within nanoparticles is a promising approach of advanced technology in sustainable agriculture. Lambda-cyhalothrin (LC) was encapsulated by the ionotropic gelation technique into chitosan (CS)/tripolyphosphate (TPP) and CS/alginate (ALG) matrixes. CS-LC nanoparticles were characterized, and their efficacy was then evaluated against the key pest of soft fruits in Europe and the United States, *Drosophila suzukii*. The encapsulation efficiency (74%), nanoparticle yield (80%), polydispersity index (0.341), zeta potential (−23.1 mV) and particle size (278 nm) were determined at the optimum conditions. FTIR confirmed the cross-linkage between CS and TPP/ALG in the nanoparticles. The optimum formula recommended by the fractional factorial design was associated with the formulation variables of CS of high or low molecular weight, cross-linking agent (TPP), LC concentration (1.5% *w/v*) and stirring rate (1500 rpm), showing the highest desirability value (0.5511). CS-LC nanoparticles of the lowest particle size (278 nm) exhibited the highest percent mortality of *D. suzukii* males (86%) and females (84%), exceeding that caused by the commercial product (Karate-zeon^®^ 10% CS) at 2 HAT. This is the first work to use the ionic gelation technique to make LC nanoparticles, to the best of our knowledge. The encapsulation of chemical pesticides within biodegradable polymeric nanoparticles could be helpful for establishing a sustainable IPM strategy with benefits for human and environmental health and the lifetime of pesticides.

## 1. Introduction

Agricultural production is hampered by a large number of variable insect pests, which cause losses of about 18–20% of annual crop production worldwide, valued at more than USD 470 billion [1]. At present, chemical control is still the main control method used for managing these damaging pests [2,3,4,5,6,7]. However, the increased use of chemical pesticides on agricultural crops has raised a great number of economic, ecological and health concerns, leading to the development of pest resistance, the elimination of pests’ natural enemies—as well as other beneficial species—and environmental pollution [8,9,10,11,12,13,14].

Pyrethroids are synthesized as derivatives of the botanical pyrethrin [15,16]. More than 520 tons of pyrethroids are used annually in the pest control worldwide [17]. They have become the main chemical class used in agricultural, veterinary and household pest management [18]. Moreover, they play a vital role in public health. Pyrethroids are most commonly used for mosquito net control due to their rapid effect at low application rates and their relative safety for human contact and household usage [19,20]. However, their continuous application has caused adverse effects on the environment, human health and beneficial microorganisms [15]. They can cause changes in the plasma biochemical profile and increase SGPT activity in humans [15]. Lambda-cyhalothrin (LC) is one of the most common pyrethroids used for agricultural and household pest control [19,20]. It is a wide-spectrum pyrethroid [21], acting through contact and stomach action on the nervous system [22]. However, LC is a very poorly water-soluble insecticide (5 μg/L at 21 °C) [23].

Given this limited water solubility [24], nanotechnology may offer effective solutions to overcome such drawbacks [25]. Producing nano-formulations able to decrease hazards to the end user and at the same time be efficient in pest control has captured the imagination of researchers, manufacturers and even the general population [26]. Recently, delivery systems have emerged as a promising option for pesticide delivery [26,27,28]. Chitosan (CS), a synthetic polymer, is one of the most promising polymers used for efficient delivery of agrochemicals because of its proven biocompatibility, biodegradability, non-toxicity and adsorption properties [29,30]. Agrochemicals encapsulated in CS matrices have the ability to function as a protective reservoir for the active ingredients [29]. They showed major advantages over traditional methods, including facilitating the uptake of active ingredients through the cell membrane, protecting ingredients from the surrounding environment and controlling pesticides’ release [29].

The ionotropic gelation technique is the most important technique for ionic cross-linking of CS with low molecular weight, hydrophobic and high molecular weight ions [31,32]. It is an electrostatic interaction between differently charged products, one of which at least is a polymer, under mechanical stirring [33,34]. The most commonly used cross-linking agents in this technique are sodium tripolyphosphate (TPP) and alginate (ALG) [33,34,35]. However, there is still a wide knowledge gap regarding its potential application in the encapsulation of active ingredients of pesticides in agriculture [29].

The present study aimed to prepare CS nanoparticles loaded with LC by the ionotropic gelation technique and to evaluate the bioactivity of the prepared CS-LC nanoparticles against a newly emerged destructive insect, the cherry fly, *Drosophila suzukii* Matsumura (Diptera: *Drosophilidae*), commonly known as the spotted wing drosophila (SWD). It is a highly polyphagous invasive pest, native to Asia, which has recently caused significant damage to a wide variety of berry and stone fruit crops in the United States [36,37,38], South America [39], Canada [40] and Europe [41,42]. The SWD’s polyphagia, the infestation during the ripening stage close to harvest, the length and scale of the harvesting period and the presence of different varieties of its host vary greatly, complicating its chemical control [43]. An adulticide–ovicide and residual approach is needed because effective control of larvae is not possible [6,44]. In Europe and the United States, broad-spectrum insecticides (e.g., organophosphates, carbamates, pyrethroids, spinosyns and diamides) have been shown to be effective [6,45,46,47,48,49,50]. In this effort, it is important to reduce the amount of used pesticides while maintaining their effectiveness in order to limit the residues on fruits [51] and the development of insecticide resistance in the SWD [42,52].

## 2. Materials and Methods

### 2.1. CS-LC Nanoparticles

#### 2.1.1. Materials

Lambda-cyhalothrin 97% technical grade was purchased from Orient Resources International Co., Ltd. (ORICO), Zhuhai, Guangdong, China. Chitosan of high and low molecular weights, sodium tripolyphosphate, sodium alginate and Tween 80 were purchased from Sigma-Aldrich, Milan, Italy. Karate-zeon^®^ 10% CS was provided by Syngenta, Milan, Italy. Sucrose was obtained from El-Gomhoria Co., Alexandria, Egypt. All used reagents were of analytical grade.

#### 2.1.2. Experimental Design

An experimental 2^4−1^ fractional factorial design was tailored to evaluate and optimize the effect of the formulation variables on the physicochemical properties of the CS-LC nanoparticles [53]. The fractional factorial design reduced considerably the number of preparations (from 16 preparations to 8, in the present case of four factors at two levels each), which is economic and time saving [54]. The four factors were CS molecular weight (MW) type, cross-linking agent, pesticide concentration and stirring rate, each at two levels (low and high) as shown in Table 1. The compositions of the designed formulations are shown in Table 2.

#### 2.1.3. Preparation of CS-LC Nanoparticles

The nanoparticles were prepared using the ionotropic gelation technique previously described in [55,56,57,58]. Firstly, CS (high or low molecular weight) at 0.4% *w/v* was dissolved in 1% *v/v* acetic acid solution and kept overnight at room temperature to allow amino group formation [59,60,61]. Tween 80 (1% *v/v*) was added into the solution as a surfactant to reduce the nanoparticles’ hydrodynamic diameter [61]. The core material (LC powder) was added to CS solutions under magnetic stirring until homogenous CS-LC dispersions were obtained. Cross-linking agent solutions of TPP and ALG were separately prepared in distilled water [60]. Nanoparticles were formed instantaneously upon the dropwise addition of the cross-linking solution to CS-LC dispersions under continuous gentle stirring for 60 min. Sucrose (5% *w/v*) was added to the prepared nanosuspension to act as a cryoprotectant. The nanoparticles were separated by cooling centrifugation at 20,000 rpm at 4 °C for 30 min, followed by freeze-drying at 20 Pa and −50 °C for 24 h (Freeze-drier Alpha 1–2 LD Martin-Christ-Germany).

### 2.2. Characterization of Nanoparticles

#### 2.2.1. Physical Properties

##### Yield Percentage, Pesticide Loading and Entrapment Efficiency

The lyophilized LC-CS nanoparticles were weighed and referenced against the weight of the initial components according to the following equation:

(1) $$\mathrm{Yield}\;\mathrm{percentage}\;(\%\mathrm{YP})=\frac{\mathrm{Weight}\;\mathrm{of}\;\mathrm{nanoparticles}}{\mathrm{Total}\;\mathrm{initial}\;\mathrm{solids}\;\mathrm{weight}}\times 100\;$$

The percent pesticide loading (%PL) refers to percentage of pesticide relative to amount of solid polymer, calculated as follows:

(2) $$\%\mathrm{PL}=\frac{\mathrm{Amount}\;\mathrm{of}\;\mathrm{pesticide}\;\mathrm{entrapped}\;}{\mathrm{Total}\;\mathrm{weight}\;\mathrm{of}\;\mathrm{polymer}\;\mathrm{incorporated}}\times 100\;$$

The percentage of entrapment efficiency (%EE) was determined by the direct method previously described in [62]. Five milligrams of CS-LC nanoparticles were dissolved in 20 mL of 1% *v/v* acetic acid and kept in an incubator overnight to allow for complete dissolution of CS. The precipitated LC residue was firstly separated by decantation of the supernatant CS solution and then dissolved in 10 mL of methanol. The concentration of LC was measured spectrophotometrically at λ_max_ 278 nm (Spectrophotometer, Jasco, Tokyo, Japan). The %EE was then calculated using the following equation.

(3) $$\%\mathrm{EE}=\;\frac{\mathrm{Amount}\;\mathrm{of}\;\mathrm{LC}\;\mathrm{actually}\;\mathrm{present}\;}{\mathrm{Total}\;\mathrm{amount}\;\mathrm{of}\;\mathrm{LC}\;\mathrm{added}}\times 100\;$$

##### Particle Size, Polydispersity Index and Zeta Potential

Based on the dynamic light scattering technique, the particle size, polydispersity index (PDI) and zeta potential of the developed formulas were measured by photon correlation spectroscopy (Zetasizer, Malvern Panalytical Ltd., Malvern, UK). A sample of the developed nanoparticles was mixed with 10 mL distilled water. All the samples were sonicated for 5 min prior to determination. All measurements were taken as an average of a triplicate.

#### 2.2.2. Morphological and FTIR Examination

Transmission electron microscope (TEM; JEOL, JEM-1230, Akishima, Tokyo, Japan) analysis was performed to examine the morphological characteristics of the LC nanoparticles. The samples were diluted appropriately with 0.1 M phosphate buffer to capture the particles’ image. The structural features of nanoparticles were measured by a Fourier transform infrared (FTIR) spectrometer (FTIR- 4100^®^, Jasco, Tokyo, Japan using KBr pellets [55].

### 2.3. In Vitro Release Study

The release of LC from the developed nanoparticles was tested in distilled water, using a modified USP apparatus-I dissolution tester (Hanson research SR6 dissolution tester, Hanson Research Corp., Chatsworth, CA, USA). A specified weight of each sample equivalent to 5 mg LC was placed in 100 mL dissolution medium at room temperature, 25 °C, and a stirring rate of 50 rpm. At predetermined time intervals, 1 mL of dissolution medium was withdrawn and replaced by the same volume of fresh media. The concentration of LC in the collected samples was measured spectrophotometrically at λ_max_ 278 nm. The release study was performed in triplicate for each sample for a period of 6 h.

To examine the kinetics of LC release from the prepared nanoparticles, the release data were fitted to models representing zero-order, first-order and Higuchi’s models. The correlation coefficient (R^2^) values were calculated from the plots of Q vs. t for zero-order, log (Q_0_–Q) vs. t for first-order and Q vs. t^1/2^ for Higuchi; Q is the amount of pesticide released at time t, and (Q_0_–Q) is the amount of the pesticide remaining after time t [63,64].

### 2.4. Bioactivity of Nanoparticles

#### 2.4.1. Laboratory SWD Colony

*Drosophila suzukii* used in the experiments originated from wild specimens from Northern Italy collected in the autumn of 2016. Mixed-sex adults were placed in 50 mL plastic culture vials (diameter 30 mm, length 115 mm) with ∼15 mL specific medium for *D. suzukii* rearing (Bloomington Drosophila Stock Centre, Indiana University) [5,48,49,50]. Cultures were maintained in climate chambers at 23 ± 1 °C, 70 ± 10% R.H. and 16:8 h L: D regime. Wild *D. suzukii* adults were introduced into the colony on multiple occasions in 2017 and 2018 to ensure that the genetic make-up of the individuals screened in the laboratory was representative of the field population.

#### 2.4.2. Laboratory Bioassays

Karate-zeon^®^ (lambda cyhalothrin 10% *w/v*) was applied as a standard at its recommended label rate of application (25 mL h L^−^^1^). For accurate comparison, the amount from each formulation containing 10% LC *w/v* was calculated according to the %PL of each formula. Ten treatments including the eight developed formulations, Karate-zeon and control (water) were evaluated. The experiment was arranged in a randomized complete block design, with 10 replicates for each treatment. A dipping non-infested fruit bioassay was used following the method described in Shawer et al. (2018) [49], Cuthbertson, et al. [65]. Strawberries used for the bioassay were collected from insecticide-free orchards located in Verona province, Northeast Italy. Strawberries were previously tested for pesticide residues by exposing some fruit samples to adult SWDs for 24 h (h), and those with evidence of active pesticide residue (>5% mortality after 24 h exposure) were discarded. The fruits were completely dipped in the treatments for 30 s, dried and placed into 10 cm diameter ventilated plastic pots. Ten *D. suzukii* flies (five males + five females) were introduced into each plastic pot containing treated fruits. The pots were then maintained throughout the experimental period under controlled environmental conditions (23 ± 1 °C, 70% RH and 16 L: 8 D photoperiod). The mortality of *D. suzukii* adults was recorded at 4 and 16 h after treatment.

### 2.5. Statistical Analysis

Data of adult mortality were statistically analyzed by the generalized linear model (GLM) using one-way analysis of variance (ANOVA) followed by means separation with Tukey’s least significant difference (LSD) test using JMP software version 4.0.4 (SAS Institute Inc., Cary, NC, USA). The percentages of SWD adult mortality were transformed to arcsine (sqrt (%mortality)) before analysis to stabilize variance, and reported means were back-transformed to percentages for presentation. Differences were considered significant at α = 0.05.

## 3. Results

### 3.1. Characterizations of Nanoparticles

#### 3.1.1. Physical Properties

The %yield ranged from 50 to 80% for all preparations (Table 3). ANOVA results confirmed that the designed formulation variables showed a significant effect (*p* < 0.01) on the yielded nanoparticles of all formulations. All formulations prepared with the high stirring rate (1500 rpm) showed higher yield percentages than those prepared with the low stirring rate (500 rpm).

The %PL and %EE of the prepared formulations were significantly affected by the molecular weight of CS (*p* = 0.0046) (Table 3). The formulations prepared with high MW CS cross-linked with either TPP or ALG showed higher %PL and %EE than those formulated with low MW CS. An inverse relationship between LC concentration and %PL or %EE was shown, except the case of CS with low MW/TPP (F3 and F4).

**Figure 1:**
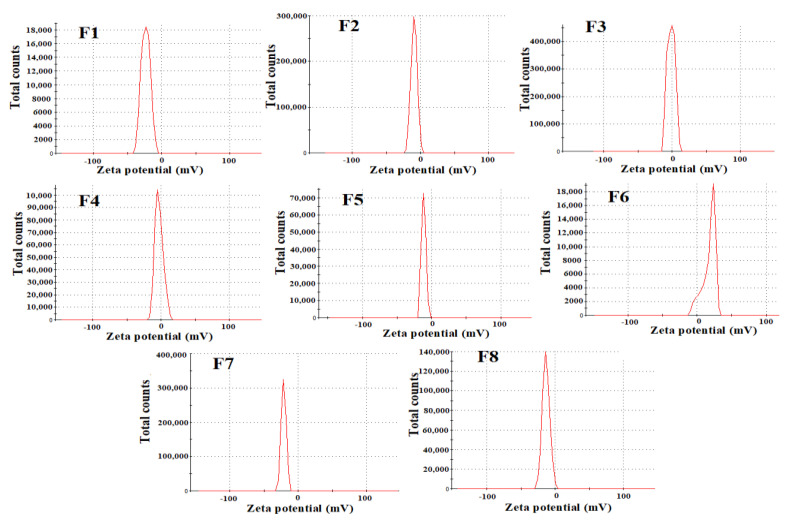
Zeta potentials of prepared LC-CS nanoparticles.

The respective average diameters of LC-loaded CS nanoparticles ranged from 278 to 415.7 nm (Table 3 and Supplementary Figure S1). The formula coded F7 (low MW CS cross-linked with ALG under low stirring at 500 rpm) showed the least particle size (278 nm) among the formulations. Formulas F7 and F8, both prepared by CS with low MW cross-linked with ALG, showed the lowest nanoparticle sizes (278 and 289 nm, respectively). The results showed low significant effect for the formulation variables on the particle size of LC-CS nanoparticles (*p* = 0.057). However, ANOVA showed a significant effect of CS MW and LC concentration on PDI. All formulations showed PDI ≤ 0.5, indicating homogeneity and a narrow range of distribution between particles. The results (Figure 1) demonstrated zeta potentials ranging between −2.3 and −23.1 mV. The formulas coded F1, F7, F6 and F2 showed the highest zeta potentials (>−19). F4 and F3 showed low zeta potentials (<5.7).

Overall, both F1 and F3 showed the highest desirability, yielding the same value of 0.5511 (Table 3). They were followed by F2 (0.5506), F4 (0.5410), F7 (0.4967), F5 (0.4801), F6 (0.4668) and F8 (0.4665). These results indicate the recommendation of F1 and F3 as optimum pesticide formulations. Moreover, the formulations F1, F3, F2 and F4 showed the four highest desirability values (≥0.5410).

#### 3.1.2. Morphological Characterization

**Figure 2:**
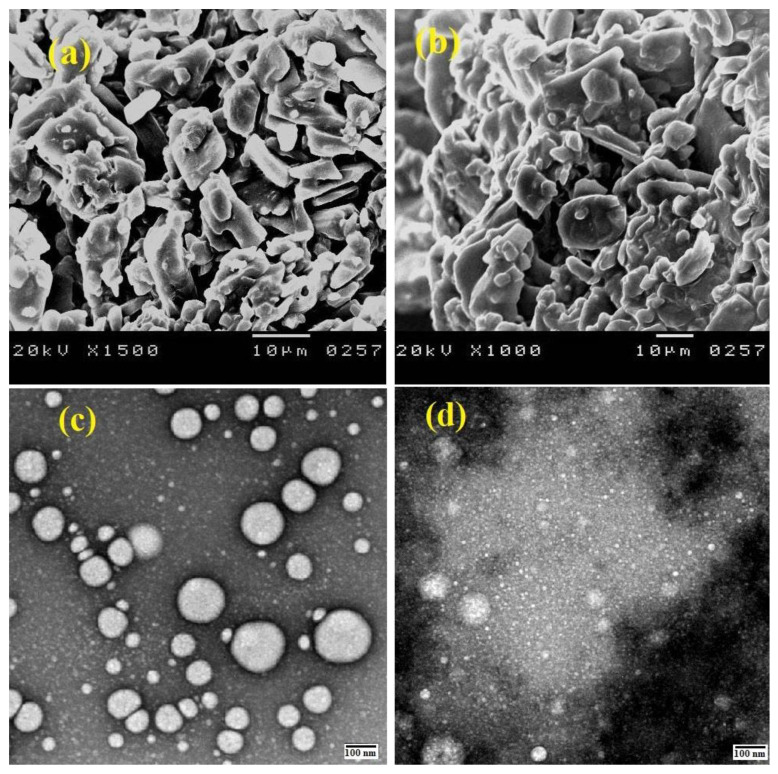
SEM and TEM images for LC-CS nanoparticles cross-linked with TPP (**a**,**c**) and TEM and SEM images (**b**,**d**) for LC-CS nanoparticles cross-linked with ALG.

In the present study, SEM and TEM images have shown the morphological properties and surface appearance of the fabricated nanoparticles. Figure 2 shows the SEM and TEM images of F3 and F5 as models for LC-CS nanoparticles cross-linked with TPP and ALG, respectively. The obtained nanoparticles had nearly spherical shape and a smooth surface, with a wide range of particle sizes (90–400 nm).

#### 3.1.3. FTIR Analysis

**Figure 3:**
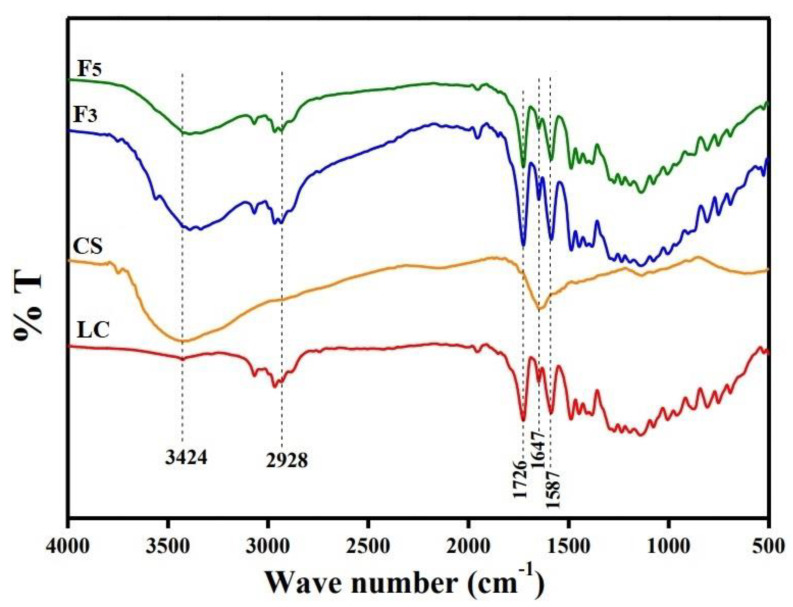
FTIR spectra of LC, CS, CS/TPP-loaded LC nanoparticles (F3) and CS/ALG−loaded LC nanoparticles (F5).

**Figure 4:**
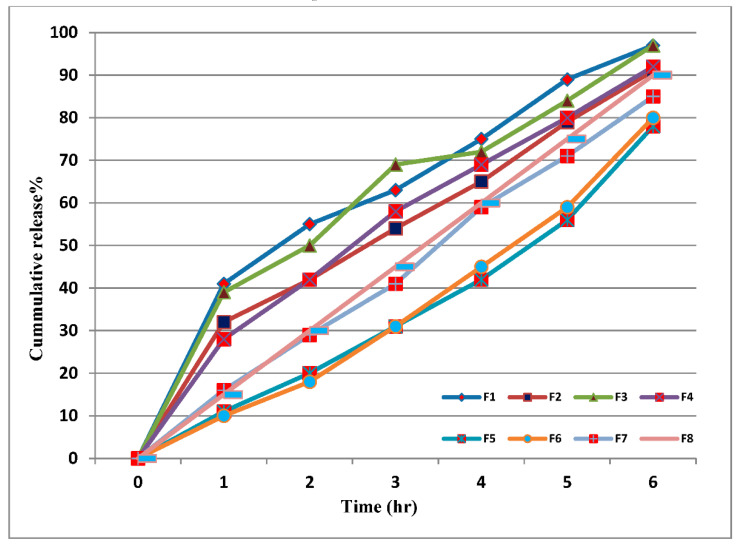
LC release profile from prepared LC-loaded CS nanoparticles (F1 to F8).

The FTIR spectra of CS, LC and LC-loaded CS nanoparticles were studied (Figure 3). Regarding the spectrum of LC, the peak appearing at 3068 cm^−^^1^ is attributed to C-H bonds in the aromatic ring; however, C-H bonds in the aliphatic chain presented at 2900–3000 cm^−^^1^, and the sharp peak at 1726 cm^−^^1^ is assigned to the C=O of LC; another two peaks at 1647 and 1587 cm^−^^1^ are attributed to C=C groups [66]. Furthermore, the peaks at 1077 and 800 cm^−^^1^ are ascribed to the CF_3_ and V-Cl groups of LC. In the CS spectrum, the strong and wide peak at 3424 cm^−^^1^ is attributed to the O-H stretching vibration, while the peak at 1635 cm^−^^1^ is assigned the CONH_2_ group, and the peak at 2928 cm^−^^1^ corresponds to the CH_2_ group. In the spectrum of CS-TPP nanoparticles (F3), the tip of the peak at 3424 cm^−^^1^ of the CS spectrum was shifted to a lower wavenumber. Also, the peak for the N-H bending vibration of the amide II carbonyl stretch at 1650 cm^−^^1^ in CS was shifted and overlapped with the peak of the C=O bond of LC at 1586.5 cm^−^^1^. These results can be attributed to the linkage between phosphoric and ammonium ions. In the ALG/CS nanoparticles spectrum (F5), the peak for the N-H bending vibration of the amide II carbonyl stretch at 1647.8 cm^−^^1^ in CS of high MW was shifted and overlapped with the peak of the C=O bond of LC at 1586.5 cm^−^^1^; this can be attributed to CS and ALG cross-linking. Overall, the characteristic peaks of LC were not affected by encapsulation in CS nanoparticles, as shown in Figure 4, indicating high compatibility between LC and other excipients. The obtained FTIR spectra of LC in the lower region (<3000 cm^−1^) are similar, yet a few prominent changes were observed in the higher region (>3000 cm^−1^). The broad bands at 3426 cm^−1^ were flattened and shifted to 3390 and 3391 cm^−1^, respectively, in F3 and F5. These results confirm the successful cross-linking of CS nanoparticles as well as the successful loading of LC into CS.

### 3.2. In Vitro Release Study

The release profile of LC-loaded CS nanoparticles cross-linked with TPP exhibited an initial burst release of about 30–40% in the first hour compared to only 10–15% drug release from ALG-cross-linked nanoparticles. However, our observations showed that about 90% of the loaded LC was released within 6 h of incubation in distilled water for all formulas (Figure 4). However, the release profile of LC-loaded CS nanoparticles cross-linked with ALG showed a constant sustained release of the pesticide during the time of the release study.

All the release profiles fit the zero order, which proved the ability of the nanoparticles to encapsulate LC and retard its release until sustainability was reached. The release of LC following the zero order may be due to the diffusion of encapsulated pesticide from the nanoparticle after erosion of its wall by the medium.

### 3.3. Bioactivity of Nanoparticles

All prepared formulations and Karate-zeon^®^ significantly caused *D. suzukii* mortality higher than the control at 2 HAT (male: F = 12.91, DF = 9, *p* < 0.0001; Female: F = 14.82; DF = 9; *p* < 0.0001) and at 16 HAT (males: F = 327.26, DF = 9, *p* < 0.0001; females: F = 180.90; DF = 9; *p* < 0.0001) (Table 4). The results revealed that the prepared formulations provided percent adult mortality higher than Karate-zeon^®^ at 2 HAT (except F4 and F5 in females) and at 16 HAT (with statistical differences in females). The highest mortality was exhibited by F3 and F7, with both showing low particle sizes (304 and 278 nm, respectively) among the formulations; therefore, it could be concluded that there is a relationship between the particle size and the biological performance of the prepared nano-formulations.

## 4. Discussion

In the present study, we encapsulated lambda-cyhalothrin in a CS-TTP/ALG matrix using the ionic gelation technique. Based on our knowledge, this is the first study to prepare LC nanoparticles with the ionic gelation technique. These results open the door to the possibility of preparing other pyrethroids as nano-pesticides via this technique. The formed cross-linking matrix controls the release of the pesticide’s active ingredients. This conception was confirmed by Shen, et al. [67], who recorded significant differences in the release properties of LC nanoparticles. However, LC was previously prepared as a nano-pesticide using other methods such as the solvent evaporation and melt emulsification–high-pressure homogenization techniques. For example, Shen et al. [67] prepared LC nanoparticles by the solvent evaporation method utilizing polylactic acid as a carrier. They produced uniform-spherical and smooth-surface nanoparticles with a size of less than 200 nm. An acceptable LC-loading capacity (46.6%) and a high encapsulation efficiency (more than 90%) were obtained [67]. Pan, et al. [68] prepared LC 5% as a nanosuspension by the melt emulsification–high-pressure homogenization method, obtaining a mean particle size of 16.01 ± 0.11 nm. In 2019, an LC nanosuspension was prepared by the one-step melt emulsification technique [69]. The produced particles had excellent properties with the smaller particle size (12.0 ± 0.1 nm) the researchers obtained.

We used a fractional factorial design to select the most suitable preparation variables to obtain the optimum formulation in terms of chemical and physical properties. It recommended the optimum formulas (with F1 and F3 giving the highest desirability values). The common formulation variables between both these formulations were the cross-linking agent (TPP), LC concentration (1.5% *w/v*) and stirring rate (1500 rpm). Moreover, the principle stable variable between the four formulations (F1, F3, F2 and F4) with the highest desirability values (≥0.5410) was the cross-linking agent, TPP. These data reveal that TPP was more suitable than ALG as a cross-linking agent for preparing CS-LC nanoparticles. However, the presence of two polymers (CS and ALG) may have facilitated interaction, resulting in greater %EE [70]. The TEM photographs confirmed that loaded nanoparticles had nearly a uniform spherical shape and a smooth surface [67]. The FTIR spectra confirmed the cross-linkage between CS and the cross-linking agents (TPP or ALG). The stirring speed during ionic gelation significantly affected the reaction yield [71]. The nanoparticle sizes and PDI were significantly affected by the CS molecular weight [72]. The formulations prepared using low-MW CS had smaller particle sizes than these obtained with high-MW CS. This may be attributed to the low viscosity of the low-MW CS used [72]. The prepared nanoparticle formulations provided activity against *D. suzukii* adults greater than that caused by a commercial product (Karate-zeon^®^) containing same active ingredient (LC).

The obtained results indicate that the bioactivity of LC nanoparticles against *D. suzukii* adults was affected by their particle size. The best activity of the prepared LC nanoparticles was caused by the formula with the smallest particle size (278 nm). Our results are in harmony with these previously obtained in [23,73,74,75,76,77]. Those findings have the potential to be very beneficial and applicable in the control of the SWD insect, which attacks fruits nearing harvest during the ripening period, when the use of chemical insecticides is critical. In such cases, reducing the pesticide application times is strongly encouraged in order to avoid exceeding the maximum residue limits on fruits. This can be accomplished by improving the biological performance of the insecticides used, reducing application times and chemical residues on fruits, lowering the negative effects on the environment and non-targeted organisms and delaying the development of insecticide resistance. Increasing the surface area and, therefore, the solubility, are two well-known benefits of using smaller particles [78,79]. Wang et al. [77] previously demonstrated that avermectin nano-delivery systems can significantly improve pesticides’ controllable release, photostability and biological activity, thereby improving efficiency and reducing pesticide residues. The ionic gelation approach was used by Rajkumar et al. [76] to encapsulate peppermint oil in chitosan nanoparticles. It was reported that using nanoparticles considerably enhanced the oil’s toxicity against the stored product pests *Sitophilus oryzae* (L.) and *Tribolium castaneum* (Herbst). In vivo, nanoparticles inhibited AChE activity in *S. oryzae* and *T. castaneum* by 52.43 and 37.80%, respectively, under optimal conditions. Paulraj et al. [75] developed CS-TPP nanoparticles loaded with a botanical pesticide, Ponneem^®^, a blend of seed oils of *Pongamia pinnata* and *Azadirachta indica*, and assessed its antifeedant, larvicidal and growth-regulation effects against *Helicoverpa armigera*, an important lepidopteran pest. The size of the nanoparticles (32 to 90 nm) was confirmed by electron micrography, and the cross-linking of chitosan with TPP was confirmed by FTIR spectroscopy. The nanoparticles evidenced 88.5 and 90.2% antifeedant and larvicidal activity against *H. armigera*, respectively. In addition, they reduced the weights of *H. armigera* pupae substantially.

The ability of the prepared nanoparticles to retard the release of LC in the agricultural environment was assessed by conducting a release study. The release profile of LC-loaded CS nanoparticles cross-linked with TPP exhibited an initial burst release of about 30–40% in the first hour, followed by a controlled release of 50–60% for the subsequent 5 h. The observed burst effect can be attributed to dissociation of LC molecules that were loosely bound to the surface of CS nanoparticles [55,80]. The second part of the release profile is related to the slow release of entrapped LC molecules at an approximately constant rate that arises from the slow degradation of the nanoparticles [55,81]. However, the release profile of LC-loaded CS nanoparticles cross-linked with ALG showed a constant sustained release of LC during the release study. This release behavior may be due to the high density of the nanoparticle core and also an increase in the diffusional path length, which the pesticide molecules have to traverse. Grillo, et al. [82] formulated chitosan nanoparticles loaded with the herbicide paraquat by modifying CS with TPP via the ionic gelation technique. Their kinetic release study revealed that the nanoparticles delayed the release time of paraquat compared to the free herbicide. Silva, et al. [83] also noted that nanoparticles prolonged the release of paraquat for 2 h longer than the free herbicide and attributed this to the strong interaction of paraquat with the nanoparticles, which could have an inhibitory effect on the release of the herbicide into the soil.

In summary, ionic gelation could be a potential method for preparing nanoparticles of LC or other pyrethroids for application in pest management, particularly in the home and in relation to public health. Nanoparticles, on the other hand, should be studied more thoroughly in terms of their environmental impact.

## 5. Conclusions

It could be concluded that there is a significant relationship between the particle size and the biological performance of the nano-formulations. The encapsulating of chemical pesticides in biodegradable polymers such as chitosan is a promising approach in sustainable agriculture due to its improving their potential activity in target pests, reducing concentration or application times and/or rates of used chemicals and in turn decreasing the toxicity to human, non-targeted organisms and the risk of wider environmental contamination.

## Figures and Tables

**Table 1:** Factors and levels of factorial design.

Independent Variables	Levels	
	Low	High
CS type (0.4% *w/v*)	Low MW	High MW
Cross-linking agent	TPP (0.3% *w/v*)	ALG (0.2% *w/v*)
LC concentration (% *w/v*)	1	1.5
Stirring rate (rpm)	500	1500

**Table 2:** Compositions of lambda-cyhalothrin nanoparticles.

Formula Code	CS MW	Cross-Linking Agent (% *w/v*)	Pesticide Conc (% *w/v*)	Stirring Rate (rpm)
F1	High	TPP (0.3)	1.5	1500
F2	High	TPP (0.3)	1	500
F3	Low	TPP (0.3)	1.5	1500
F4	Low	TPP (0.3)	1	500
F5	High	Alginate (0.2)	1.5	1500
F6	High	Alginate (0.2)	1	500
F7	Low	Alginate (0.2)	1.5	1500
F8	Low	Alginate (0.2)	1	500

**Table 3:** Physical characterization of CS-LC nanoparticles and desirability values showing the optimum formula.

Formula	%Yield	%PL	%EE	Particle Size (nm)	PDI	Z-Potential (mv)	Desirability
F1	79.64 ± 2.1	10.86 ± 0.3	53.12 ± 3.5	346.1 ± 5.59	0.528 ± 0.05	−23.1 ± 0.5	0.5511
F2	58.44 ± 3.1	10.15 ± 0.9	68.93 ± 2.9	414.3 ± 14.2	0.448 ± 0.08	−9.13 ± 0.3	0.5506
F3	61.62 ± 1.7	9.8 ± 0.1	46.53 ± 3.9	308.4 ± 19.5	0.341 ± 0.03	−1.21 ± 0.3	0.5511
F4	57.31 ± 2.0	3.9 ± 0.2	24.95 ± 1.3	363 ± 27.7	0.457 ± 0.06	−2.84 ± 0.3	0.5410
F5	68.55 ± 3.2	8.41 ± 0.1	59.56 ± 2.1	353.3 ± 24.9	0.390 ± 0.05	−11.3 ± 0.2	0.4801
F6	57.48 ± 1.9	10.72 ± 0.4	73.56 ± 4.3	415.7 ± 22.8	0.447 ± 0.05	−19.8 ± 0.6	0.4668
F7	65.60 ± 2.5	4.15 ± 0.3	29.32 ± 1.2	278 ± 15.1	0.472 ± 0.01	−21.1 ± 1.2	0.4967
F8	59.29 ± 4.0	5.31 ± 0.1	36.12 ± 1.1	289 ± 19.1	0.453 ± 0.03	−13.2 ± 0.5	0.4665

**Table 4:** Percent mortality *(±standard deviation)* of male and female *D. suzukii* exposed to residual strawberries treated with CS-LC nanoparticles. Means within each column (time-gender) followed by the same letter (s) are not significantly different (Tukey’s LSD test; *p* = 0.05).

Formula	Percent Mortality			
	2 h		16 h	
	Male	Female	Male	Female
F1	74.0 ± 5.8 a	78.0 ± 8.7 ab	100 ± 0.0 a	98.0 ± 2.0 a
F2	74.0 ± 7.9 ab	86.0 ± 5.2 a	100 ± 0.0 a	100 ± 0.0 a
F3	84.0 ± 6.0 ab	79.0 ± 7.0 ab	100 ± 0.0 a	100 ± 0.0 a
F4	82.0 ± 6.3 ab	70.9 ± 6.3 ab	100 ± 0.0 a	100 ± 0.0 a
F5	70.0 ± 9.1 ab	60.0 ± 10.3 b	100 ± 0.0 a	100 ± 0.0 a
F6	68.0 ± 9.0 ab	80.0 ± 4.2 a	100 ± 0.0 a	100 ± 0.0 a
F7	86.0 ± 4.3 a	84.0 ± 5.0 a	100 ± 0.0 a	100 ± 0.0 a
F8	78.0 ± 6.3 ab	68.0 ± 7.4 ab	100 ± 0.0 a	100 ± 0.0 a
Karate-zeon	64.0 ± 7.8 b	74.0 ± 6.0 ab	98.0 ±2.0 a	90.0 ± 5.4 b
Control	2.0 ± 2.0 c	0.0 ± 0.0 c	12.0 ± 4.4 b	10.0 ± 3.3 c

## Data Availability

Not applicable.

## Supplementary Materials

The following supporting information can be downloaded at: https://www.mdpi.com/article/10.3390/nano12183110/s1, Figure S1: Respective average diameters of LC-loaded CS nanoparticles.
