# Supplementary figures and images for: Preparation of Lambda-Cyhalothrin-Loaded Chitosan Nanoparticles and Their Bioactivity against Drosophila suzukii
Source: Nanomaterials (Basel). 2022 Sep 8;12(18):3110. doi: 10.3390/nano12183110 (PMC9503733; doi:10.3390/nano12183110)

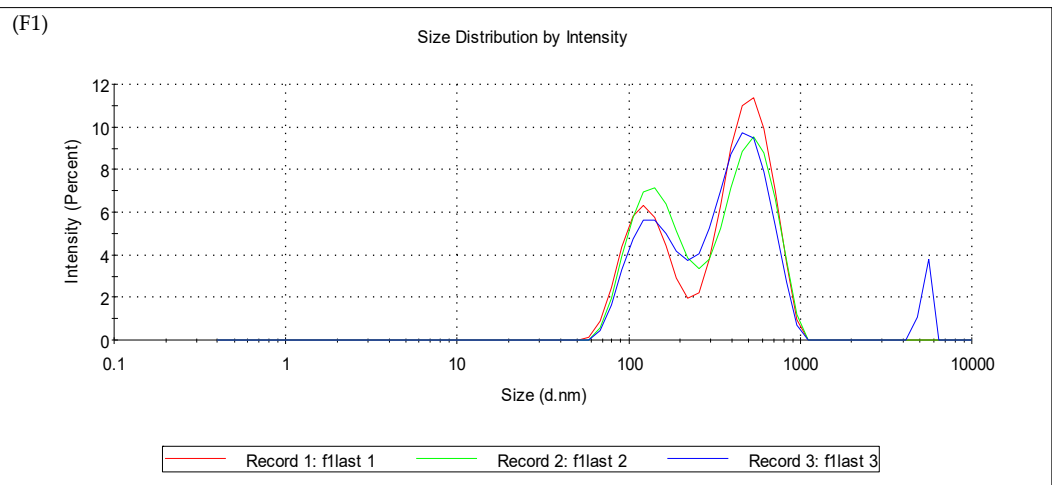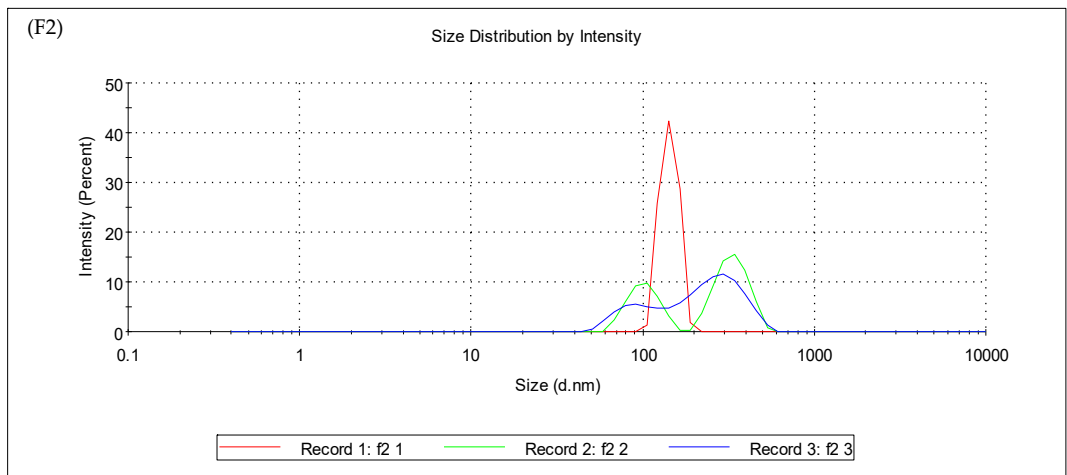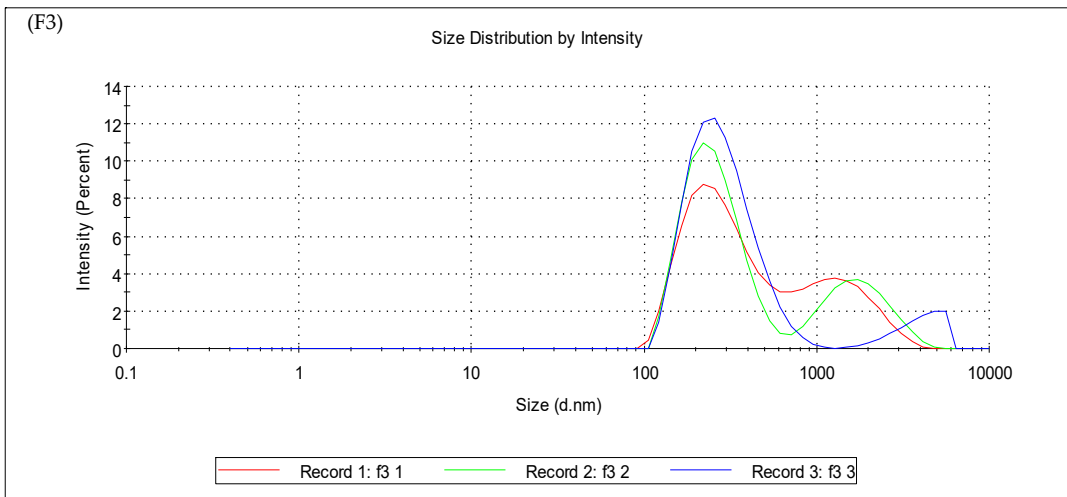

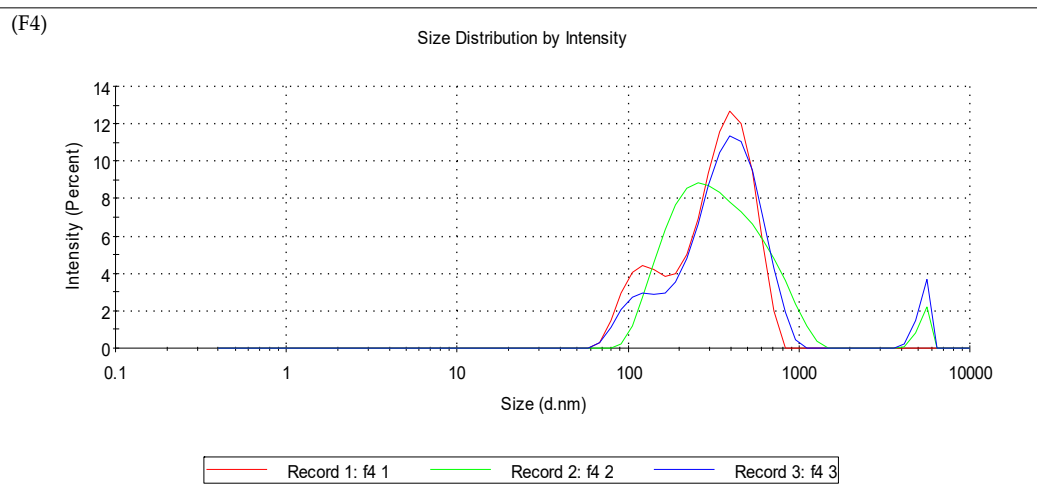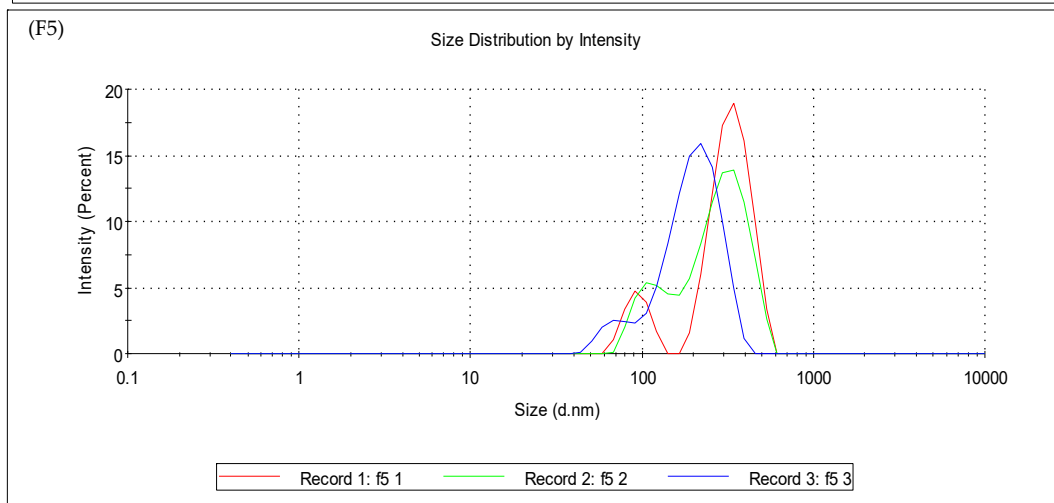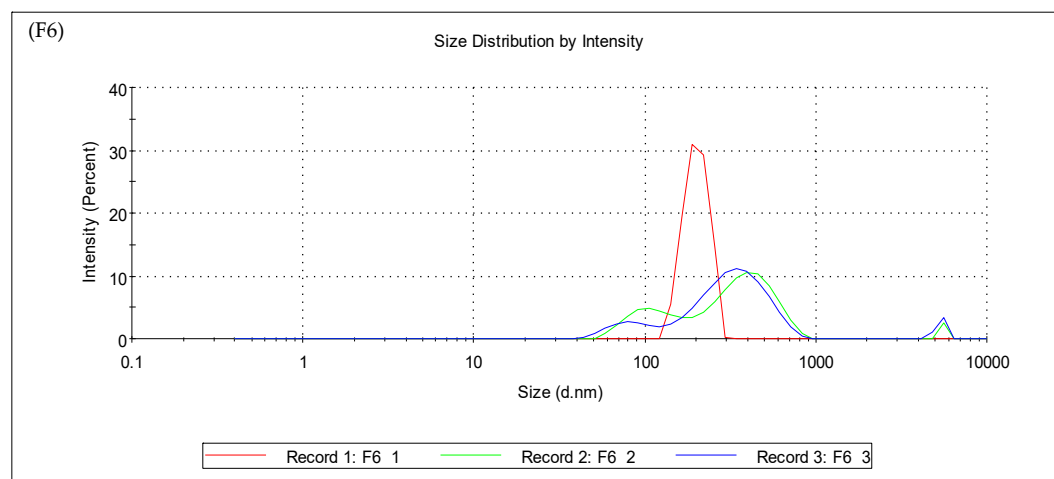

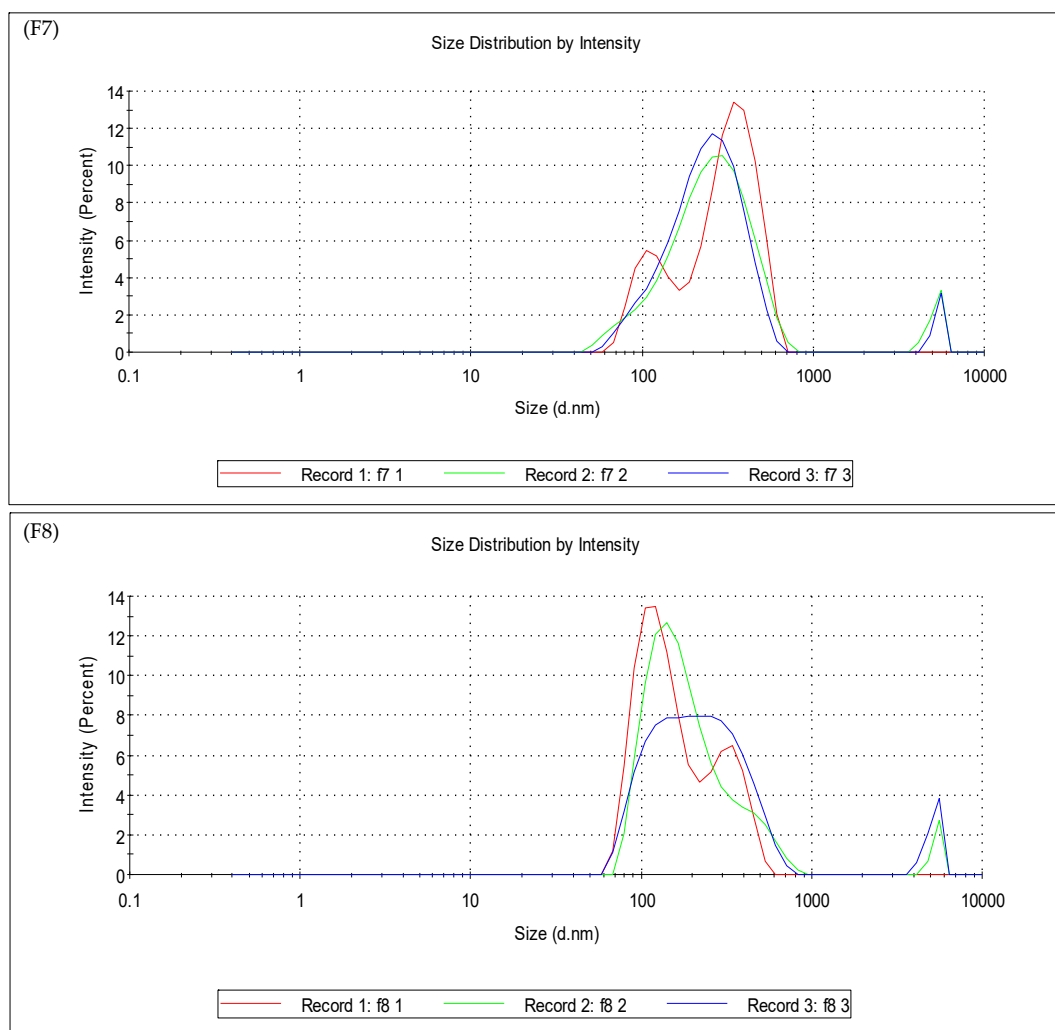

**Figure S1.** Respective average diameters of LC-loaded CS nanoparticles.

Supplement: Supplementary file 1 [file nanomaterials-12-03110-s001.zip › nanomaterials-1850821-supplementary.pdf]
